# A custom force plate for quantifying the force applied by the finger during smartphone usage

**DOI:** 10.3389/fbioe.2026.1685410

**Published:** 2026-02-17

**Authors:** Yumou Han, Filippo M. Vallerini, Fred Holdsworth, Kiatbodin Wanglertpanich, Angela E. Kedgley, Spyros D. Masouros

**Affiliations:** 1 Department of Bioengineering, Imperial College London, London, United Kingdom; 2 The Imperial College Advanced Hackspace, Imperial College London, London, United Kingdom

**Keywords:** biomechanics, crosstalk, custom force plate, kinetic, smartphone, strain gauge

## Abstract

**Introduction:**

The increase in thumb activity due to smartphone use in recent years may be associated with an elevated risk of developing musculoskeletal disorders. Prior studies on hand biomechanics during touchscreen use have indicated that activities such as swiping and tapping lead to varying levels of muscle activation and ranges of motion. Currently, however, there is no device that can be used readily to measure finger forces accurately during smartphone use.

**Method:**

This study presents the design of a portable force plate specifically developed to quantify fingertip forces during smartphone use. The device utilises a load-cell structure and foil strain gauges to measure applied force magnitude, direction, and location.

**Results:**

The device achieved a force sensitivity of 0.15 N and a positional sensitivity of 2.5 mm, with a maximum measurable force capacity of 3 N.

**Discussion:**

The portable force plate enables the study of hand kinetics whilst allowing for physiological kinematics during smartphone use, with applications spanning musculoskeletal and finite-element model development of the hand, ergonomic risk assessment, smartphone interface evaluation, and musculoskeletal injury prevention.

## 1 Introduction

In recent years, smartphones have become an integral part of daily life, introducing new functional roles for the hand, particularly for the thumb. Traditionally, the primary role of the thumb has been to grip or stabilise the hand whilst the other fingers perform desired tasks such as writing and gripping (Karakostis et al., 2021). However, the widespread adoption of smartphones has added a new dimension to thumb activity: approximately 84% of the global population use smartphones, with 61% of young adults (under 25 years) reporting smartphone addiction (Ratan et al., 2022). As a result, the thumb is now frequently engaged in activities such as swiping and tapping, requiring a large range of motion (ROM) and repetitive movement patterns.

This increase in thumb activity due to smartphone use has raised concerns about the increased risk of developing musculoskeletal (MSK) disorders, as repetitive, non-ergonomic hand and finger movements are known risk factors for conditions such as osteoarthritis and overuse syndrome (Palmer, 2003; Punnett and Wegman, 2004). Prior studies on hand biomechanics during touchscreen use have indicated that activities such as swiping and tapping lead to varying levels of muscle activation and ranges of motion (Gustafsson et al., 2018; Huang et al., 2024). Notably, among touchscreen devices, smartphones pose a particularly high ergonomic risk. Unlike larger interfaces such as tablets or information kiosks that are usually operated with both hands, smartphones are typically held and controlled with one hand. This single-handed use imposes distinct thumb and wrist kinematics and increased mechanical loading compared with two-handed operations (Ko et al., 2016; Trudeau et al., 2016). Moreover, musculoskeletal symptoms associated with us of a handheld device have been reported to be more prevalent during one-handed smartphone interactions than during the use of larger, two-handed devices (Bendero et al., 2017; Zhu and Li, 2016). However, there are limited studies that have quantified the forces exerted on the fingers during these activities.

Biomechanical studies often use MSk and finite-element (FE) models to explore the behaviour of human joints at risk of MSK disorders. These models require kinematic and kinetic data, typically obtained through motion-capture systems and force plates, respectively. However, collecting kinetic data for fingers is challenging. Conventional commercial force plates for biomechanics are primarily designed for recording ground-reaction forces in gait analysis; these are too large and lack the necessary sensitivity for studying forces applied by the hands during fine motor tasks.

As an alternative to conventional force plates, several studies have employed commercial six-degree-of-freedom (6-DOF) load cells to measure fingertip forces during touchscreen use, reporting values ranging from 0.5 N to 2.6 N (Asakawa et al., 2017; Kim et al., 2019). While informative, these approaches have limitations. Kim et al. (2019) attached the load cells to the back of the phone, thus increasing the thickness of the device, altering the physical dimensions; this likely affects hand kinematics. Asakawa et al. (2017) fixed a tablet to a rigid platform, therefore restricting posture and movement of the participants’ hand. These examples illustrate a key limitation of 6-DOF sensors in handheld applications, whereby their physical bulk and mounting requirements often compromise the physiological user behaviour. Moreover, because a single sensor only reports the resultant force and moment at one point, at least three non-co-linear load cells are required to measure both the fingertip force and its point of application (Wang et al., 2019). Six-DOF load cells are also expensive, typically costing at the order of USD 3,000–5,000 (Cao et al., 2021; Pérez Ubeda et al., 2018).

To address these limitations, this study presents a novel, custom-designed force plate capable of measuring tri-axial fingertip forces with positional information. Unlike existing commercially available load cells, the proposed device preserves the size, weight, and form factor of a conventional smartphone, thereby minimising alterations to natural interaction with the smartphone and thus hand kinematics and kinetics. Custom force plates have been designed before successfully that are suitable for capturing small-magnitude forces. An example is in the field of insect biomechanics where the most common configuration employs orthogonally arranged strain gauges on miniaturised force plates, allowing directional forces to be reconstructed via calibration matrices derived from sensor voltage outputs, with crosstalk minimised through structural optimisation and regression-based correction (Heglund, 1981). This approach has been used successfully to measure ground reaction forces in geckos (Dai et al., 2011), ants (Reinhardt and Blickhan, 2013), and earthworm (Fang et al., 2024), with micronewton to millinewton resolution. Building on these principles, the proposed force plate enables high-sensitivity force measurements suitable for capturing the low-magnitude, dynamic forces applied by the fingers in smartphone activities such as tapping and swiping. Research tools for studying biomechanics and ergonomics, such as musculoskeletal and finite-element models, require accurate force inputs. The proposed force-plate outputs, combined with kinematic measurements from well-established commercial motion-capture systems, will enable the study of ergonomics and biomechanics of the hand during smartphone use.

## 2 Materials and methods

### 2.1 Force plate design

To replicate the size and feel of a real smartphone, the force plate was designed to match the dimensions of a commercial device. Dimensions of 109 smartphones were sourced from three major manufacturers: Samsung, Apple, and Xiaomi. The iPhone 8, being approximately 10th percentile in size, was chosen as the starting point for this study. Upon successful implementation, any design of this size can be readily scaled to a larger size.

**FIGURE 1:**
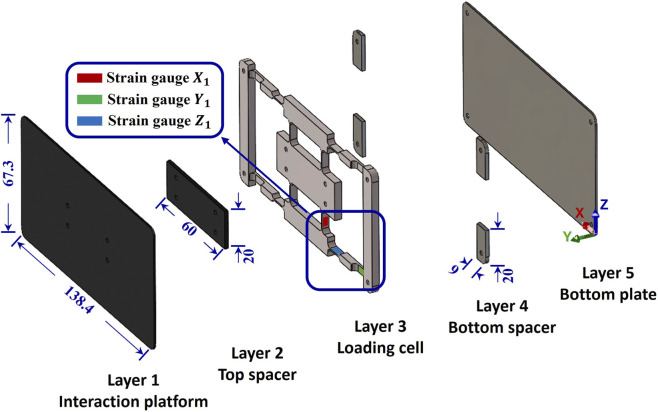
Design of force plate assembly also showing the positions of three of the strain gauges, $X_{1}$ , $Y_{1}$ and $Z_{1}$ , in one-quarter of the plate. All dimensions shown are in mm.

The force plate assembly was designed in SolidWorks (v2023, Dassault Systèmes, United States). It comprised five layers, whereby the loading cell was sandwiched between four components (Figure 1; Table 1). The loading cell featured 12 spring beams; a strain gauge (350 Ω foil strain gauges, RunesKee, Shenzhen City Xinglinlieren Technology Co., Ltd., China) was mounted on each. The 350 Ω resistance was selected over the 120 Ω to reduce current flow and thereby minimise self-heating. Each strain gauge was dedicated to capturing forces primarily in one direction of a Cartesian coordinate system that was aligned with the major axes of the assembly (Figure 1). As the resistance change of each strain gauge was small, the output voltage was amplified twice with a gain of 2000. To assess and correct for potential voltage drift due to temperature, the circuit was tested by running it for 30 min, monitoring the shift in output voltage. With five repeated tests, an increase of 0.0177 ± 0.0106 V was observed in the first 15 min, and the voltage remained stable thereafter, with a shift of 0.0013 ± 0.0032 in the second 15 min. A resistor was also added to the circuit to zero the voltage at the start of use, eliminating any resistance changes caused by storage conditions.

**TABLE 1:** Dimensions (mm) of the force plate assembly.

Layer	Layer 1 Interaction platform	Layer 2 Spacer	Layer 3 Loading cell	Layer 4^a^ Spacer	Layer 5 Bottom plate
Width (mm)	67.3	20	67.3	9	67.3
Length (mm)	138.4	60	138.4	20	138.4
Thickness (mm)	1	2	4	2	1.2

^a^ Layer 4 consists of 4 identical sections; the dimensions shown are for one piece.

The interaction platform, where the fingers interact with the force plate during use, mimicked the touchscreen of the smartphone. Spacers were placed on either side of the loading cell to ensure that any force applied to the interaction platform was transferred fully to the central area of the loading cell. Only the two ends of the loading cell were supported, preventing the central portion from contacting the bottom plate. The gaps between the spacers allowed effective cable routing.

The interaction platform and adjacent spacers were made from carbon fibre, which was chosen for its lightweight properties and high stiffness. The loading cell was made from polycarbonate, which undergoes elastic deformation under typical touchscreen forces, ranging from 0.5 N to 3.5 N. The bottom plate and adjacent spacers were made from G304 stainless steel to add weight and enhance the stability of the assembly. The total weight of this design was 145.3 g, closely matching the weight of the iPhone 8 (148 g).

**FIGURE 2:**
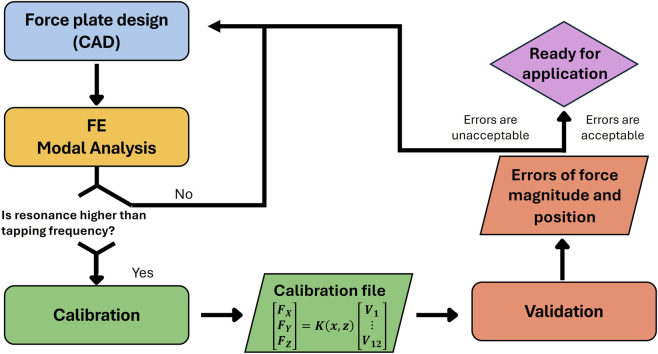
Flowchart of the method for force plate design, calibration, and validation.

In order to assess the ability of the force plate to meet its intended accuracy in recording force magnitude and location, (a) a modal finite-element analysis was conducted to ensure that natural frequencies would not affect the recordings, (b) calibration to link strain-gauge outputs with force magnitude and location was carried out, and (c) test for validation - confirmation of the accuracy of the force plate’s recordings - was conducted whereby the force plate recordings were compared with those of a uniaxial testing machine. This process is illustrated in a flowchart (Figure 2).

### 2.2 Structural and modal analysis

To aid in the design of the force plate, an FE model was developed in Abaqus (v2020, SIMULIA, Dassault Systèmes, France). The FE model had two objectives: the first was to verify that the strain in the spring beam is sufficiently large and exhibits a linear relationship with the applied force and position; and the second was to analyse the natural frequency to ensure that resonance does not compromise the measurements of the force plate. The model only incorporated the interaction platform, top spacer and loading cell; the bottom spacer and bottom plate were simplified as fixed boundary conditions since they only provided support and were expected to not deform during use. Four central holes on layers 1, 2 and 3 were connected as rigid bodies, and the contact between each layer was modelled as smooth (no friction) and hard (no penetration). The model was meshed with 20-node quadratic brick elements with reduced integration (type C3D20R). A sensitivity analysis determined that a 0.5 mm element-edge size was sufficient, whilst a finer mesh was applied to the spring beams, where strain gauges were to be mounted, ensuring a minimum of four finite elements through the thickness. All materials were assumed to be linearly elastic and isotropic; (G304) stainless steel: Young’s modulus, E = 190 GPa, Poisson’s ratio ν = 0.27; carbon fibre: E = 243 GPa, ν = 0.27; polycarbonate: E = 2.4 GPa, ν = 0.36.

Smartphone activities, such as typing, involve repetitive interaction with the touchscreen. To ensure that resonance would not affect the performance of the force plate, its natural frequency needed to be compared with the typical frequency of smartphone users touching the screen. Modal analysis was conducted to determine the natural frequency of the force plate. Since no existing literature provided data on typing frequency, ten frequent smartphone users, each with a screen time of over 4 hours per day, were recruited. They were asked to type a message as quickly as possible, and their frequency was calculated by dividing the number of letters typed by the time taken in seconds. This part of the study received ethical approval from the Research Governance and Integrity Team (Imperial College Research Ethics Committee Reference Number: 6455822).

### 2.3 Calibration

Force plate calibration is necessary to determine the linear matrix transformation (Equation 1; Equation 2) that relates strain-gauge outputs to the force vector applied to the interaction platform (Dai et al., 2011; Reinhardt and Blickhan, 2013; Schrand, 2007).

$$\left[\begin{array}{c} F_{X}\\ F_{Y}\\ F_{Z} \end{array}\right]=K\left(x,z\right)\left[\begin{array}{c} V_{1}\\ \vdots \\ V_{12} \end{array}\right]$$ (1)

$$\left[\begin{array}{c} V_{1}\\ \vdots \\ V_{12} \end{array}\right]={\left[V_{i}\right]}_{i=1}^{12}=K^{-1}\left[\begin{array}{c} F_{X}\\ F_{Y}\\ F_{Z} \end{array}\right]$$ (2)

where $F_{X}$ , $F_{Y}$ and $F_{Z}$ are the three components of the applied force, $V_{i}$ is the voltage of strain gauge i ; and K is the characteristic matrix; the inverse of K ( $K^{-1}$ ) is termed the sensitivity matrix.

Although each spring beam was designed to respond primarily to forces in its designated axis direction, crosstalk was inevitable. Crosstalk was accounted for by treating the output voltage of each strain gauge as a combination of three components, reflecting the influence of forces from different directions (Equation 3). $K^{-1}$ was obtained through regression analysis of the applied load magnitude and position against the output voltage of strain gauges, which was expressed in terms of x and z (Equation 4).

$$V_{i}=\left(\gamma_{iX}z+c_{iX}\right)F_{X}+\left(\alpha_{iY}x+\gamma_{iY}z+c_{iY}\right)F_{Y}+\left(\alpha_{iZ}x+c_{iZ}\right)F_{Z}$$ (3)

$$K^{-1}={\left[\begin{array}{ccc} \gamma_{iX}z+c_{iX} & \alpha_{iY}x+\gamma_{iY}z+c_{iY} & \alpha_{iZ}x+c_{iZ} \end{array}\right]}_{i=1,\ldots ,12}$$ (4)

Coefficients $\alpha_{iY}$ , $\alpha_{iZ}$ , $\gamma_{iX}$ , $\gamma_{iY}$ , $c_{iX}$ , $c_{iY}$ , and $c_{iZ}$ were associated with strain gauge i and can be determined through calibration tests. Specifically, regression analyses were conducted between the applied forces and output voltages, followed by a second regression analysis between the positions of load application and output voltages. The coefficients were derived from the results of these regression analyses.

**FIGURE 3:**
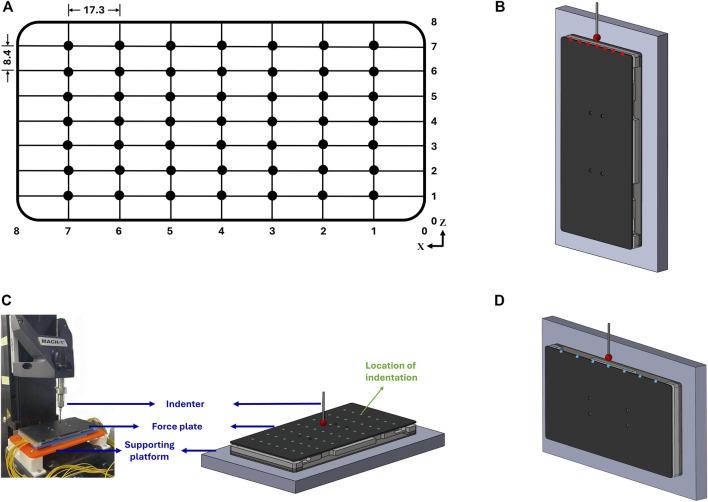
**(A)** Calibration grid on interaction platform. Calibration in **(B)** X direction, **(C)** Y direction, and **(D)** Z direction. Calibrations were run by applying a force of 0.5–3 N in each of X, Y, and Z directions at each position indicated by red, green and blue dots respectively on the interaction platform. All dimensions shown are in mm.

To establish the relationship between the output voltage of strain gauges and the magnitude and position of an applied force, forces were applied separately in each of X, Y, and Z directions at regularly spaced positions on the interactive platform. A materials testing machine (MACH-1, Biomomentum Inc., Laval, Canada), equipped with a 10 N single-axis load cell and a 5 mm indenter (Figure 3) was used to apply forces in a grid pattern (Figure 3A). Forces ranging from 0.5 N to 3.5 N were applied in the X direction at seven positions (Figure 3B); in the Y direction (normal to the interaction platform) at 49 positions (Figure 3C); and in the Z direction at seven positions (Figure 3D). This force range was based on the reaction forces observed during touchscreen use (Asakawa et al., 2017; Kim et al., 2019). The strain-gauge output voltage was collected using the cRIO-9048, module NI-9205, and LabVIEW (National Instrument, Texas, United States). The data were post-processed and visualised in MATLAB (v.R2024b, MathWorks, Massachusetts, United States).

Solving an algebraic system of five-variable nonlinear equations with quadratic terms requires only five independent equations to determine the variables ( $F_{X}$ , $F_{Y}$ , $F_{Z}$ , x and z ). In this study, each strain gauge provides one independent equation; therefore, although 12 strain gauges were installed, only five were needed and used to solve for the unknown coefficients. The combination of the five strain gauges with the highest coefficient of determination ( $R^{2}$ ) was selected to calculate the force’s magnitude and position. The correct solution was identified by constraining the range of $F_{X}$ , $F_{Y}$ , $F_{Z}$ , x and z , as well as cross-checking the results with solving using other five-strain gauge combinations. The redundancy of the strain gauges enabled cross-validation of the results, optimization of the gauge combination, and accounted for potential gauge failures or malfunctions, ensuring reliable measurements.

### 2.4 Accuracy

The MACH-1 system was used to assess the accuracy of the force plate in measuring both the magnitude and position of applied forces.

**FIGURE 4:**
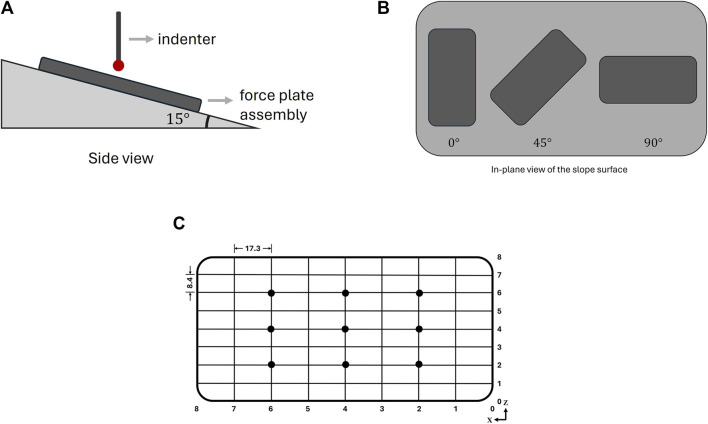
Test setup to determine load cell accuracy: **(A)** diagrammatic side view of the setup; **(B)** in-plane view of the sloped surface; **(C)** positions of load application on the surface of the interaction platform.

The bottom plate of the force-plate assembly was mounted on a sloped platform angled at 15° (Figure 4A). Within the plane of the sloped surface, the force plate was orientated at 0°, 45°, and 90° (Figure 4B), enabling simultaneous application of normal and shear forces. Static forces of 1, 2, and 3.5 N were applied at nine designated positions on the plate (Figure 4C) per orientation using a 5 mm diameter spherical indenter.

The measurement error was defined as the difference between the value measured by the MACH-1 and the value measured by the force-plate assembly. A total of 792 combinations of five strain gauges were evaluated in their ability to measure accurately magnitude and position of the applied force. The selection criteria for an optimal set focused on minimising estimation errors in the force components ( $F_{X}$ , $F_{Y}$ and $F_{Z}$ ) and position coordinates (X and Z). Combinations with force-estimation errors within ±0.15 N and positional errors in the X and Z directions within ±2.5 mm were set as thresholds. Furthermore, the overall positional deviation, defined as the absolute distance between the actual and estimated positions, was required to remain below 3 mm.

### 2.5 Dynamic validation

**FIGURE 5:**
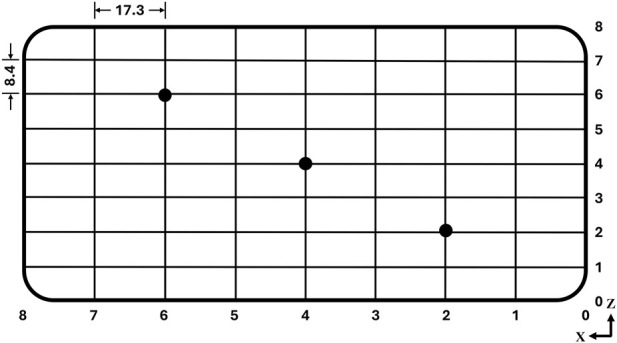
Diagram showing the three locations on the interaction platform used for the cyclic loading test.

To evaluate the stability and repeatability of the force plate under dynamic loading, a cyclic test was performed using the MACH-1 system. A sinusoidal force 3.5 N in amplitude was applied for 1,000 consecutive cycles at a frequency of 3 Hz, corresponding to the average tapping frequency during smartphone use, at three representative positions on the interaction platform (Figure 5) to account for spatial variability. The force measured by the force plate was compared with the reference force recorded by the MACH-1 system across the 1,000 cycles.

## 3 Results

### 3.1 Natural frequency

The average frequency with which participants touched the screen of their smartphones during rapid typing was 2.7 ± 0.7 Hz (mean ± SD; range 1.89–4.12 Hz). The first mode of the modal analysis was an order of magnitude higher than the frequency with which participants were tapping the screen, and so the effect of resonance on the force plate was deemed minimal (Table 2).

**TABLE 2:** Natural frequencies of the design determined by finite-element modal analysis.

Mode	Frequency (Hz)	Associated motion
1	47	Oscillatory translation along X-axis
2	52	Oscillatory translation along Z-axis
3	64	Oscillatory rotation about the X-axis
4	70	Oscillatory rotation about the Z-axis
5	80	Oscillatory rotation about the Y-axis
6	84	Oscillatory translation along Y-axis

### 3.2 Calibration

In both the experimental and FE models, the resultant strain displayed a linear relationship with force (Table 3). $K^{-1}$ was obtained through regression analysis; Equation 5 shows the $K^{-1}$ for the load case at the centre ( x=4,y=4 ).

$$\left[\begin{array}{c} \begin{array}{c} \begin{array}{c} V_{1}\\ V_{2} \end{array}\\ \begin{array}{c} V_{3}\\ V_{4} \end{array}\\ \begin{array}{c} V_{5}\\ V_{6} \end{array} \end{array}\\ \begin{array}{c} \begin{array}{c} V_{7}\\ V_{8} \end{array}\\ \begin{array}{c} V_{9}\\ V_{10} \end{array}\\ \begin{array}{c} V_{11}\\ V_{12} \end{array} \end{array} \end{array}\right]=\left[\begin{array}{ccc} \begin{array}{c} \begin{array}{c} -0.393\\ -0.524 \end{array}\\ \begin{array}{c} 0.133\\ 0.294 \end{array}\\ \begin{array}{c} -0.035\\ \begin{array}{c} -0.028\\ 0.017\\ \begin{array}{c} 0.020\\ -0.021\\ \begin{array}{c} -0.068\\ 0.006\\ 0.054 \end{array} \end{array} \end{array} \end{array} \end{array} & \begin{array}{c} \begin{array}{c} -0.033\\ -0.037 \end{array}\\ \begin{array}{c} -0.027\\ -0.023 \end{array}\\ \begin{array}{c} 0.316\\ \begin{array}{c} 0.336\\ 0.264\\ \begin{array}{c} 0.193\\ -0.070\\ \begin{array}{c} -0.207\\ -0.106\\ -0.202 \end{array} \end{array} \end{array} \end{array} \end{array} & \begin{array}{c} \begin{array}{c} -0.051\\ 0.103 \end{array}\\ \begin{array}{c} -0.021\\ 0.015 \end{array}\\ \begin{array}{c} -0.110\\ \begin{array}{c} 0.168\\ -0.073\\ \begin{array}{c} 0.026\\ -0.369\\ \begin{array}{c} 0.613\\ -0.666\\ 0.553 \end{array} \end{array} \end{array} \end{array} \end{array} \end{array}\right]\left[\begin{array}{c} F_{X}\\ F_{Y}\\ F_{Z} \end{array}\right]$$ (5)

**TABLE 3:** Regression coefficients and crosstalk of strain guages. Regression coefficient β represents the sensitivity of each strain gauge to applied forces $F_{X}$ , $F_{Y}$ and $F_{Z}$ on the centre ( x = 4, z = 4). Crosstalk (%) is calculated as the ratio of β in one direction to the sum of β across all directions, indicating the relative contribution of each force component to the total output. $R^{2}$ denotes the coefficient of determination for each regression model. NA, Not Applicable.

Strain gauge	$\beta_{F_{X}}$	Crosstalk %	${R_{F_{X}}}^{2}$	$\beta_{F_{Y}}$	Crosstalk %	${R_{F_{Y}}}^{2}$	$\beta_{F_{Z}}$	Crosstalk %	${R_{F_{Z}}}^{2}$
$X_{1}$	−0.393	NA	0.999	−0.033	6.92	0.990	−0.051	10.69	0.990
$X_{2}$	−0.524	NA	0.997	−0.037	5.57	0.996	0.103	15.51	0.933
$X_{3}$	0.133	NA	0.998	−0.027	14.92	0.999	−0.021	11.60	0.991
$X_{4}$	0.294	NA	0.996	−0.023	6.93	0.997	0.015	4.52	0.968
$Y_{1}$	−0.035	7.59	0.991	0.316	NA	0.994	−0.110	23.86	0.999
$Y_{2}$	−0.028	5.26	0.953	0.336	NA	0.996	0.168	31.58	0.988
$Y_{3}$	0.017	4.80	0.980	0.264	NA	0.994	−0.073	20.62	0.988
$Y_{4}$	0.020	8.37	0.991	0.193	NA	0.996	0.026	10.88	0.997
$Z_{1}$	−0.021	4.57	0.989	−0.070	15.22	0.999	−0.369	NA	0.999
$Z_{2}$	−0.068	7.66	0.998	−0.207	23.31	0.999	0.613	NA	0.996
$Z_{3}$	0.006	0.77	0.756	−0.106	13.62	0.999	−0.666	NA	1.000
$Z_{4}$	0.054	6.67	0.992	−0.202	24.97	0.997	0.553	NA	0.999

The relationship between the positions where the load was applied and output voltage of each strain gauge was expected to be linear. The coefficients of determination ( ${R_{F_{m,n}}}^{2}$ ) quantify the goodness of fit in the regression model relating output voltage to the load position, *n*, which represents a spatial variable that may vary along the X-direction, Z-direction, or across the XZ-plane, under an applied force in direction *m* (Table 4).

**TABLE 4:** Coefficients of determination, ${R_{F_{m,n}}}^{2}$ , of the regression between $\beta_{F}$ and coordinates.

Strain gauge	$\gamma_{iX}$	$c_{iX}$	${R_{F_{X,z}}}^{2}$	$\alpha_{iY}$	$\gamma_{iY}$	$c_{iY}$	${R_{F_{Y,xz}}}^{2}$	$\alpha_{iZ}$	$c_{iZ}$	${R_{F_{Z,x}}}^{2}$
$X_{1}$	0.086	−0.727	0.99	−0.033	0.012	0.042	0.89	−0.207	0.773	1.00
$X_{2}$	−0.095	−0.156	1.00	−0.041	0.005	0.109	0.97	0.225	−0.786	1.00
$X_{3}$	−0.025	0.224	0.98	0.000	−0.009	0.052	0.74	0.061	−0.260	1.00
$X_{4}$	0.055	0.043	1.00	0.024	−0.015	−0.079	0.96	−0.123	0.497	1.00
$Y_{1}$	0.018	−0.108	1.00	−0.104	−0.079	1.048	1.00	−0.027	0.219	1.00
$Y_{2}$	−0.010	0.024	0.80	−0.119	0.094	0.422	1.00	0.039	−0.318	0.99
$Y_{3}$	−0.006	0.04	0.86	0.090	−0.059	0.123	1.00	0.020	−0.008	0.97
$Y_{4}$	0.004	0.01	0.61	0.083	0.052	−0.358	0.99	−0.020	0.014	1.00
$Z_{1}$	−0.034	0.144	0.98	−0.032	0.008	0.018	0.98	0.082	−0.703	1.00
$Z_{2}$	0.053	−0.294	1.00	0.017	−0.040	−0.113	0.98	−0.108	1.087	1.00
$Z_{3}$	0.067	−0.262	1.00	0.021	0.003	−0.196	0.88	−0.113	−0.203	1.00
$Z_{4}$	−0.050	0.263	1.00	−0.022	−0.038	0.037	0.99	0.080	0.215	0.96

### 3.3 Accuracy

**FIGURE 6:**
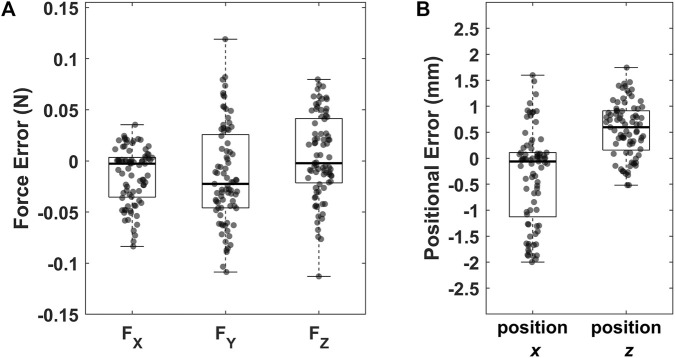
Error between **(A)** magnitude of applied and measured force; **(B)** position of applied and measured position. Outliers, defined as values below the first quartile minus 1.5 times the interquartile range or above the third quartile plus 1.5 times the interquartile range, are denoted by red crosses.

Five combinations of strain gauges were identified as meeting the accuracy criteria set. Figure 6 illustrates the error in predicting force and position using one such combination consisting of strain gauges: $X_{3}$ , $Y_{2}$ , $Y_{3}$ , $Y_{4}$ , and $Z_{4}$ . The maximum, mean, and minimum errors in the predicted forces and positions are summarised in Table 5. The remaining combinations that satisfied the selection criteria are presented in Appendix. Any of the five identified combinations were thus deemed eligible to be used for calculation the force vector.

**TABLE 5:** Maximum (max), mean, minimum (min) values and standard deviation (SD) of the error in predicting tri-axial force and position across selected strain gauge combinations.

Combination	​	$F_{x}$ (N)	$F_{y}$ (N)	$F_{z}$ (N)	x (mm)	z (mm)
$X_{2}$ , $Y_{1}$ , $Y_{2}$ , $Y_{4}$ , and $Z_{4}$	Max	0.048	0.109	0.082	1.250	2.532
	Mean	−0.012	−0.029	−0.001	−0.515	0.740
	min	−0.069	−0.108	−0.095	−2.277	−0.495
	SD	0.025	0.042	0.044	0.907	0.856
$X_{3}$ , $Y_{1}$ , $Y_{3}$ , $Y_{4}$ , and $Z_{3}$	Max	0.023	0.119	0.143	2.370	0.543
	Mean	−0.011	−0.029	0.039	−0.088	−0.096
	min	−0.059	−0.095	−0.031	−2.189	−1.363
	SD	0.023	0.042	0.050	1.456	0.347
$X_{2}$ , $Y_{2}$ , $Y_{3}$ , $Y_{4}$ , and $Z_{4}$	Max	0.016	0.121	0.080	1.618	1.638
	Mean	−0.014	−0.014	0.000	−0.375	0.576
	min	−0.065	−0.109	−0.099	−2.175	−0.279
	SD	0.021	0.048	0.044	0.937	0.502
$X_{3}$ , $Y_{2}$ , $Y_{3}$ , $Y_{4}$ , and $Z_{4}$	Max	0.035	0.119	0.080	1.597	1.742
	Mean	−0.015	−0.015	0.003	−0.374	0.581
	min	−0.084	−0.109	−0.113	−1.999	−0.256
	SD	0.028	0.049	0.043	0.890	0.510
$X_{4}$ , $Y_{2}$ , $Y_{3}$ , $Y_{4}$ , and $Z_{4}$	Max	0.036	0.121	0.123	1.562	1.668
	Mean	−0.015	−0.017	0.008	−0.333	0.527
	min	−0.132	−0.134	−0.097	−2.089	−0.224
	SD	0.036	0.052	0.048	0.914	0.469

### 3.4 Dynamic validation

**FIGURE 7:**
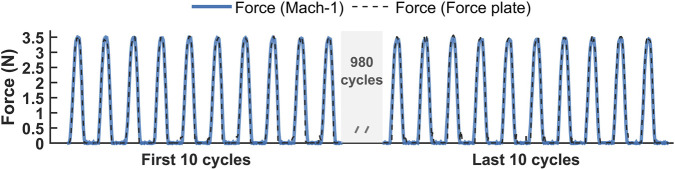
Comparison of the force recorded by the MACH-1 system (solid line) and the force measured by the force plate (dashed line) under sinusoidal loading at 3 Hz over 1,000 cycles.

Under sinusoidal loading at 3 Hz, the force measured by the force plate closely matched the reference force recorded by the MACH-1 system throughout the 1000-cycle test at each of the three loading positions. Figure 7 illustrates a representative comparison between the applied force and the force measured by the force plate at the centre ( x=4,y=4 ). Across all cycles and positions (3,000 cycles in total), the difference between the amplitude of the applied and measured force was 0.044 ± 0.036 N. Stability was further evaluated by comparing the zero-load voltage averaged over 5 s before testing with that measured over 5 s after the 1,000 loading cycle tests. The resulting voltage difference was 0.0083 ± 0.0048 V across the 12 strain gauges, indicating negligible drift.

**FIGURE A1:**
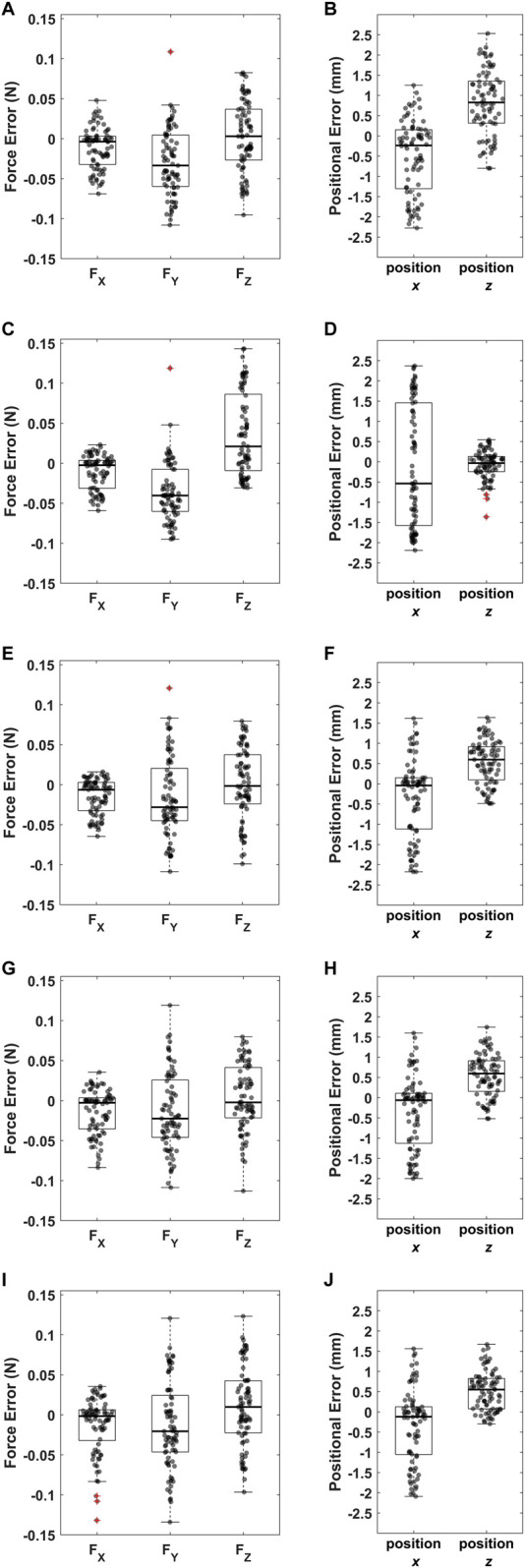
Errors between **(A,C,E,G,I)** the magnitude of the applied and measured force and **(B,D,F,H,J)** the applied and measured position for the following combinations: $X_{2}$ **,** $Y_{1}$ **,** $Y_{2}$ **,** $Y_{4}$ **,** $Z_{4}$ ; $X_{3}$ **,** $Y_{1}$ **,** $Y_{3}$ **,** $Y_{4}$ **,** $Z_{3}$ ; $X_{2}$ **,** $Y_{2}$ **,** $Y_{3}$ **,** $Y_{4}$ **,** $Z_{4}$ ; $X_{3}$ **,** $Y_{2}$ **,** $Y_{3}$ **,** $Y_{4}$ **,** $Z_{4}$ ; and $X_{4}$ **,** $Y_{2}$ **,** $Y_{3}$ **,** $Y_{4}$ **,** $Z_{4}$ . Outliers, defined as values below the first quartile minus 1.5 times the interquartile range or above the third quartile plus 1.5 times the interquartile range, are denoted by red crosses.

## 4 Discussion

This study presented the design of a force plate capable of measuring magnitude, direction and position of the force applied by fingers during smartphone use.

The high $R^{2}$ values indicate a strong linear relationship between output voltage and applied force (Table 4), confirming the effectiveness of the force-plate assembly. Crosstalk between force directions was expected and observed. When the force was applied at the centre, regression analysis (Table 4) revealed that each strain gauge was most responsive to its primary force direction. Strain gauge $X_{i}$ exhibited crosstalk from force components $F_{Y}$ and $F_{Z}$ , with crosstalk effect below 15.5%. Strain gauges $Y_{i}$ and $Z_{i}$ exhibited crosstalk between $F_{Y}$ and $F_{Z}$ , with crosstalk ranging from 10.9% to 31.6%.

Crosstalk was more pronounced under eccentric loading conditions. For example, when a force was applied at position ( X=6 , Z=4 ), $\beta_{F_{Z}}$ of strain gauge $X_{1}$ increased to 0.7, indicating a strong influence from an off-axis force component. This effect is particularly critical for force plates with a large surface area, where off-centre loads are likely and can lead to increased measurement inaccuracies. In the present design, crosstalk was not treated as noise but rather incorporated into the calibration process. Each strain gauge, although sensitive primarily to one force direction, responds to forces applied along other axes. To account for this behaviour, each gauge was calibrated under forces applied in all three directions, allowing the influence of each force component on every gauge to be quantified. The resulting sensitivity matrix therefore represents the combined effect of all directional components, enabling accurate multi-axial force reconstruction and effective compensation for crosstalk.

For the top five combinations of strain gauges used to achieve calibration, the measurement error for $F_{x}$ , $F_{y}$ and $F_{z}$ was deemed adequate for the intended application of a target force range of 0–3.5 N, with the error values within ±0.15 N. The force plate was calibrated for forces up to 3.5 N, covering the typical force range applied by fingers during smartphone use (Asakawa et al., 2017; Kim et al., 2019). The loading cell never made contact with the bottom plate for forces below 3.5 N. In additional tests, with Y forces that reached 5 N, the loading cell made contact with the bottom plate, but remained deformed within its elastic region, meaning that the maximum load limit of the load cell was not reached. As a result, the design has the potential to measure forces of up to 5 N. Further tests are required to identify the load limit of the loading cell and to ascertain whether the calibration presented here is still valid.

For position measurements, the error in the Z coordinate ranged from −1 mm to 2.5 mm, while the error in the X coordinate ranged from −2 mm to 1.5 mm. The absolute positional deviation varied between 0.3 mm and 3 mm, indicating a high level of positional accuracy, particularly when considering that the diameter of the indenter used during testing was 5 mm. Previous studies reported fingertip contact areas of approximately 60–80 mm^2^ under a normal force of approximately 0.5 N, corresponding to a contact diameter of 9 mm (Dzidek et al., 2017; Logozzo et al., 2022). Considering that the typical fingertip force during smartphone use ranges from 0.5 N to 2.6 N and that contact area at the fingertip would normally increase with load, the 0.5 N condition represents a conservative lower bound for realistic interactions. Therefore, even under this minimum loading scenario, the positional error observed in our system ( ≤ 3 mm) remains comfortably within the fingertip contact region.

The tapping frequency analysis was based on a relatively small sample size (n = 10), which may have limited the generalisability of the observed variability in tapping dynamics. The maximum measured tapping frequency (4.2 Hz), however, was one order of magnitude lower than the lowest resonance frequency of the force plate (47 Hz) and so individual variability and potential outliers present a negligible risk of interference with the force recordings.

Validation was performed under static, controlled loading conditions rather than during dynamic smartphone interactions such as tapping or swiping, which may involve transient or impact forces. Further, the maximum tested load range extended to 5 N, while calibration validity beyond 3.5 N was not conducted; further work is required to confirm linearity and structural stability for forces greater than 3.5 N. Lastly, the current prototype has not been evaluated yet for long-term durability or performance under repetitive use, which may be needed to assess when recalibration may be necessary. Addressing these aspects in future work will strengthen the robustness of the design and broaden its applicability.

The force plate presented here can now be integrated with a motion capture system to study the kinematics and kinetics of the hand when executing common smartphone activities. Such data can help smartphone design and ergonomics, such as in the study by Xiong and Muraki, (2014), whereby thumb-muscle workload varied significantly with button size and thumb movement orientation; fingertip force data from our device could directly inform considerations of interface design. Moreover, pain at the base of the thumb has been associated with prolonged smartphone use (Choi et al., 2025), highlighting the need to understand the loading response at the trapeziometacarpal joint. In biomechanical modelling, the proposed device can provide accurate force magnitude, direction, and location inputs that enable MSk and FE models of the hand to ascertain joint forces and moments and evaluate hand function during smartphone use and potential susceptibility to joint degeneration or injury. For example, realistic smartphone interaction scenarios using the force plate can explore how variations in device characteristics, such as screen size and device weight, influence fingertip forces and user biomechanics.

## Data Availability

The datasets presented in this study can be found in online repositories. The names of the repository/repositories and accession number(s) can be found in the article/Supplementary Material.

## Supplementary material

SUPPLEMENTARY TABLE 1 Electrical circuit design of the strain gauge.

SUPPLEMENTARY DATA SHEET 1 STEP files of the force-plate design.

SUPPLEMENTARY DATA SHEET 2 Error of selected strain gauge combinations (same as the supplementary figures).
