# Supplementary material for: A custom force plate for quantifying the force applied by the finger during smartphone usage
Source: Front Bioeng Biotechnol. 2026 Feb 17;14:1685410. doi: 10.3389/fbioe.2026.1685410 (PMC12953535; doi:10.3389/fbioe.2026.1685410)
Supplement: Supplementary file 1 [file Table1.docx]

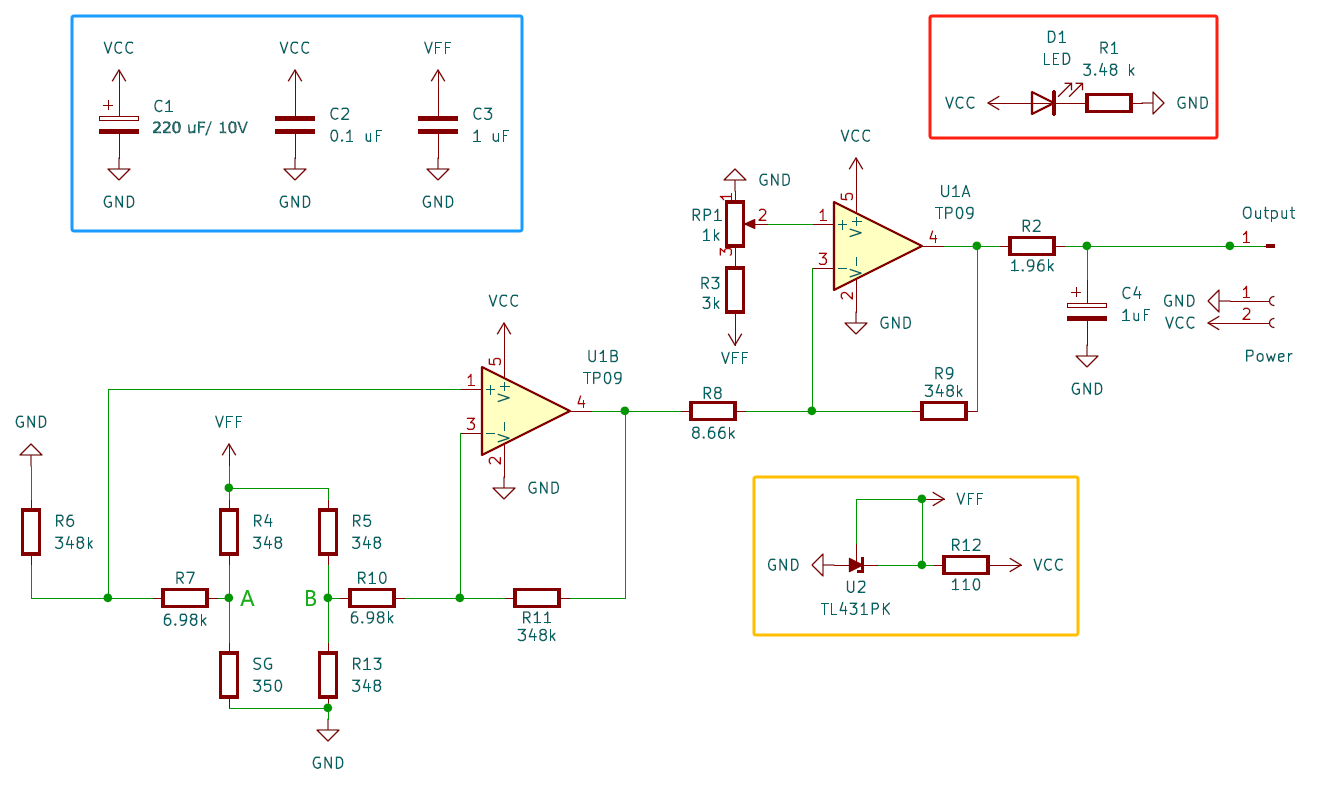


Capacitors in parallel to generate stable power supply VCC and VFF

Generate $V_{FF}$ from $V_{cc}$

$$V_{FF}=V_{cc}-I_{cc}R_{12}$$

$V_{cc}$ and $I_{cc}$ can be read from the power supply.

LED indicating if the circuit is powered

Main circuit:

$$V_{U1B,in,1}=V_{A}\frac{R_{6}}{R_{6}+R_{7}}=V_{U1B, in,3}$$

Calculate the current going through $R_{10}$

$$I_{10}=\frac{V_{B}-V_{U1B, in,3}}{R_{10}}=\frac{V_{B}-V_{A}\frac{R_{6}}{R_{6}+R_{7}}}{R_{10}}$$

Calculate the voltage drop of $R_{11}$, and $I_{10}=I_{11}$

$$\Delta V_{11}=I_{10}R_{11}=(V_{B}-V_{A}\frac{R_{6}}{R_{6}+R_{7}})\frac{R_{11}}{R_{10}}$$

$$V_{U1B,out,4}=V_{U1B, in,3}-\Delta V_{11}=V_{A}\frac{R_{6}}{R_{6}+R_{7}}-\left( V_{B}-V_{A}\frac{R_{6}}{R_{6}+R_{7}} \right)\frac{R_{11}}{R_{10}}$$

$$R_{6}=R_{11}; R_{7}=R_{10}$$

$$V_{A}\frac{R_{6}}{R_{6}+R_{7}}-\left( V_{B}-V_{A}\frac{R_{6}}{R_{6}+R_{7}} \right)\frac{R_{11}}{R_{10}}=V_{A}\frac{R_{6}}{R_{6}+R_{7}}-\left( V_{B}-V_{A}\frac{R_{6}}{R_{6}+R_{7}} \right)\frac{R_{6}}{R_{7}}$$

$$V_{A}\frac{R_{6}}{R_{6}+R_{7}}-\left( V_{B}-V_{A}\frac{R_{6}}{R_{6}+R_{7}} \right)\frac{R_{6}}{R_{7}}=\frac{V_{A}R_{6}R_{7}-V_{B}R_{6}\left( R_{6}+R_{7} \right)+V_{A}{R_{6}}^{2}}{\left( R_{6}+R_{7} \right)R_{7}}$$

$$\frac{V_{A}R_{6}R_{7}-V_{B}R_{6}\left( R_{6}+R_{7} \right)+V_{A}{R_{6}}^{2}}{\left( R_{6}+R_{7} \right)R_{7}}=\frac{V_{A}R_{6}\left( R_{6}+R_{7} \right)-V_{B}R_{6}\left( R_{6}+R_{7} \right)}{\left( R_{6}+R_{7} \right)R_{7}}$$

$$\frac{V_{A}R_{6}\left( R_{6}+R_{7} \right)-V_{B}R_{6}\left( R_{6}+R_{7} \right)}{\left( R_{6}+R_{7} \right)R_{7}}=\frac{(V_{A}-V_{B})R_{6}\left( R_{6}+R_{7} \right)}{\left( R_{6}+R_{7} \right)R_{7}}=\frac{(V_{A}-V_{B})R_{6}}{R_{7}}$$

$(V_{A}-V_{B})$ is the voltage difference caused by strain gauge deformation.

$$Gain = \frac{R_{6}}{R_{7}}=\frac{348}{6.98} \approx50$$

Look at U1A

$$V_{U1A, in,1}=\frac{{RP}_{1}}{3k+1k-{RP}_{1}}V_{FF}=\frac{{RP}_{1}}{4k-{RP}_{1}}V_{FF}=V_{U1A, in,3}$$

$$I_{8}=\frac{V_{U1B,out,4}-V_{U1A, in,3}}{R_{8}}$$

$$V_{U1A,out,4}=V_{U1A, in,3}-I_{8}R_{9}=\frac{{RP}_{1}}{4k-{RP}_{1}}V_{FF}-\frac{\frac{(V_{A}-V_{B})R_{6}}{R_{7}}-\frac{{RP}_{1}}{4k-{RP}_{1}}V_{FF}}{R_{8}}R_{9}$$

Set $\frac{{RP}_{1}}{4k-{RP}_{1}}$ a constant $n$

$$V_{U1A,out,4}=nV_{FF}-\frac{\frac{\left( V_{A}-V_{B} \right)R_{6}}{R_{7}}-nV_{FF}}{R_{8}}R_{9}=\left( 1+\frac{R_{9}}{R_{8}} \right)nV_{FF}-\frac{R_{9}}{R_{8}}\frac{R_{6}}{R_{7}}(V_{A}-V_{B})$$

$$\Delta V=\left( V_{A}-V_{B} \right)=\left( \left( 1+\frac{R_{9}}{R_{8}} \right)nV_{FF}-V_{U1A,out,4} \right)\frac{R_{8}R_{7}}{R_{9}R_{6}}=\frac{(41nV_{FF}-V_{U1A,out,4})}{2000}$$

The difference was further amplified through U1A, resulting in the total gain being the product of the gains of both amplifiers.

$$Total gain = \frac{R_{9}}{R_{8}}\frac{R_{6}}{R_{7}} \approx2000$$

Gauge factor: ${\frac{\Delta R}{R}}/\varepsilon=2.11$

$$\Delta R_{SG}=2.11\varepsilon R_{SG,0}$$

$$R_{SG}=2.11\varepsilon R_{SG,0}+R_{SG,0}=350(2.11\varepsilon+1)$$

$$V_{A}=\frac{R_{SG}}{R_{SG}+R_{4}}V_{FF}$$

$$V_{B}=\frac{R_{13}}{R_{13}+R_{5}}V_{FF}=0.5V_{FF}$$

$$V_{A}-V_{B}=\left( \frac{R_{SG}}{R_{SG}+R_{4}}-0.5 \right)V_{FF}=\left( \frac{350(2.11\varepsilon+1)}{350(2.11\varepsilon+1)+348}-0.5 \right)V_{FF}$$

$$\frac{V_{A}-V_{B}}{V_{FF}}+0.5=1-\frac{348}{350(2.11\varepsilon+1)+348}$$

$$\frac{348}{350(2.11\varepsilon+1)+348}=0.5-\frac{V_{A}-V_{B}}{V_{FF}}$$

$$\frac{348}{350(2.11\varepsilon+1)+348}=\frac{{V_{FF}-2(V}_{A}-V_{B)}}{2V_{FF}}$$

$$\frac{348}{350\left( 2.11\varepsilon+1 \right)+348}=\frac{{V_{FF}-2(V}_{A}-V_{B)}}{2V_{FF}}$$

$$348\approx350$$

$$2.11\varepsilon+2=\frac{2V_{FF}}{{V_{FF}-2(V}_{A}-V_{B})}$$

$$2.11\varepsilon=\frac{2V_{FF}}{{V_{FF}-2(V}_{A}-V_{B})}-\frac{2V_{FF}-4\left( V_{A}-V_{B} \right)}{{V_{FF}-2(V}_{A}-V_{B})}=\frac{4\left( V_{A}-V_{B} \right)}{{V_{FF}-2(V}_{A}-V_{B})}$$

$$\varepsilon=\frac{1.9\Delta V}{V_{FF}-2\Delta V}=\frac{1.9\Delta V-0.95V_{FF}+0.95V_{FF}}{V_{FF}-2\Delta V}=\frac{0.95V_{FF}}{V_{FF}-2\Delta V}-0.95$$

Since $\Delta V=\frac{(41nV_{FF}-V_{U1A,out,4})}{2000}$

$$\varepsilon=\frac{0.95V_{FF}}{V_{FF}-\frac{(41nV_{FF}-V_{U1A,out,4})}{1000}}-0.95$$
