# Supplementary figures and images for: A custom force plate for quantifying the force applied by the finger during smartphone usage
Source: Front Bioeng Biotechnol. 2026 Feb 17;14:1685410. doi: 10.3389/fbioe.2026.1685410 (PMC12953535; doi:10.3389/fbioe.2026.1685410)

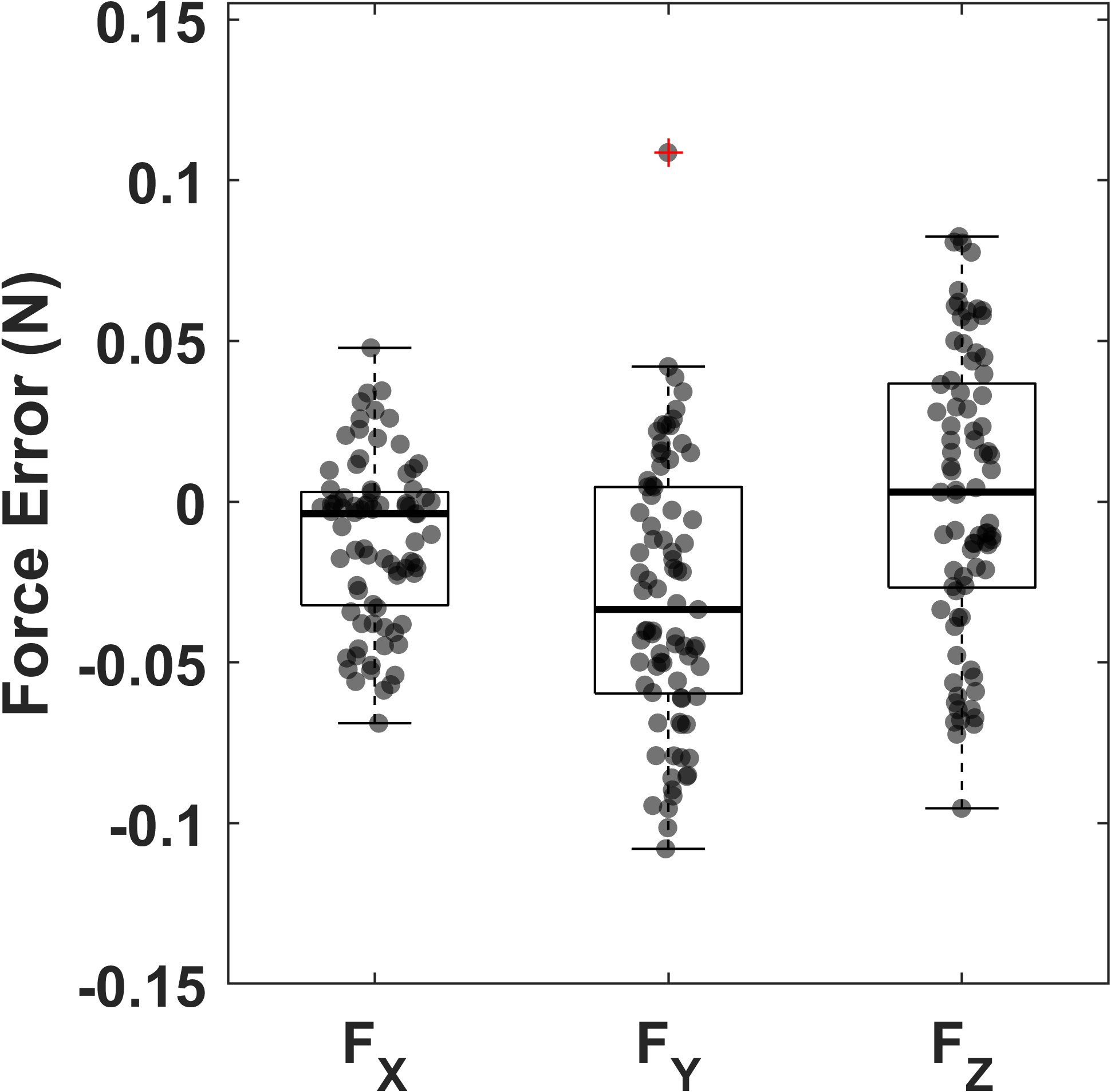

Supplement: Supplementary file 3 [file DataSheet2.zip › Appendix figures/Figure A A.jpeg]

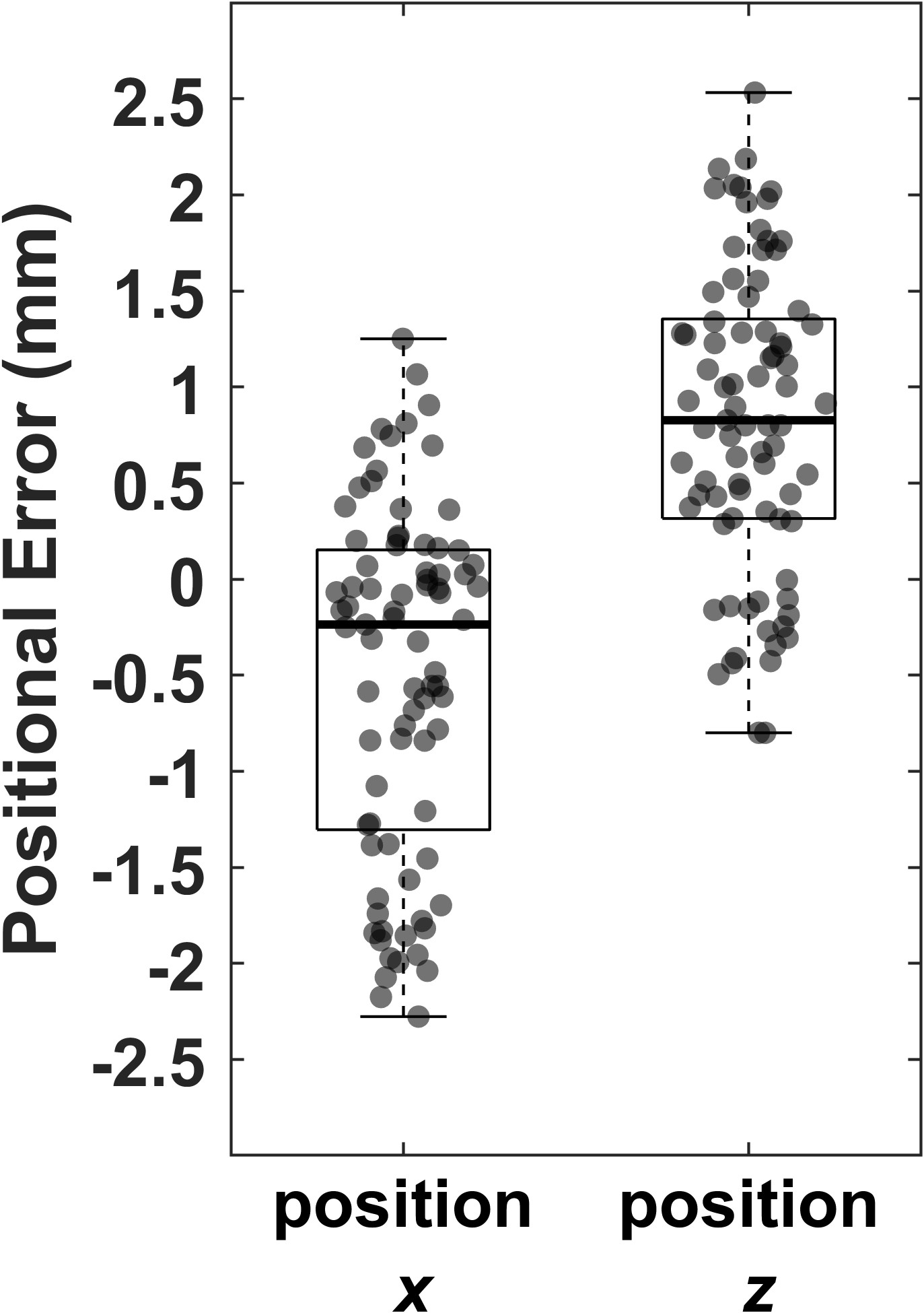

Supplement: Supplementary file 3 [file DataSheet2.zip › Appendix figures/Figure A B.jpeg]

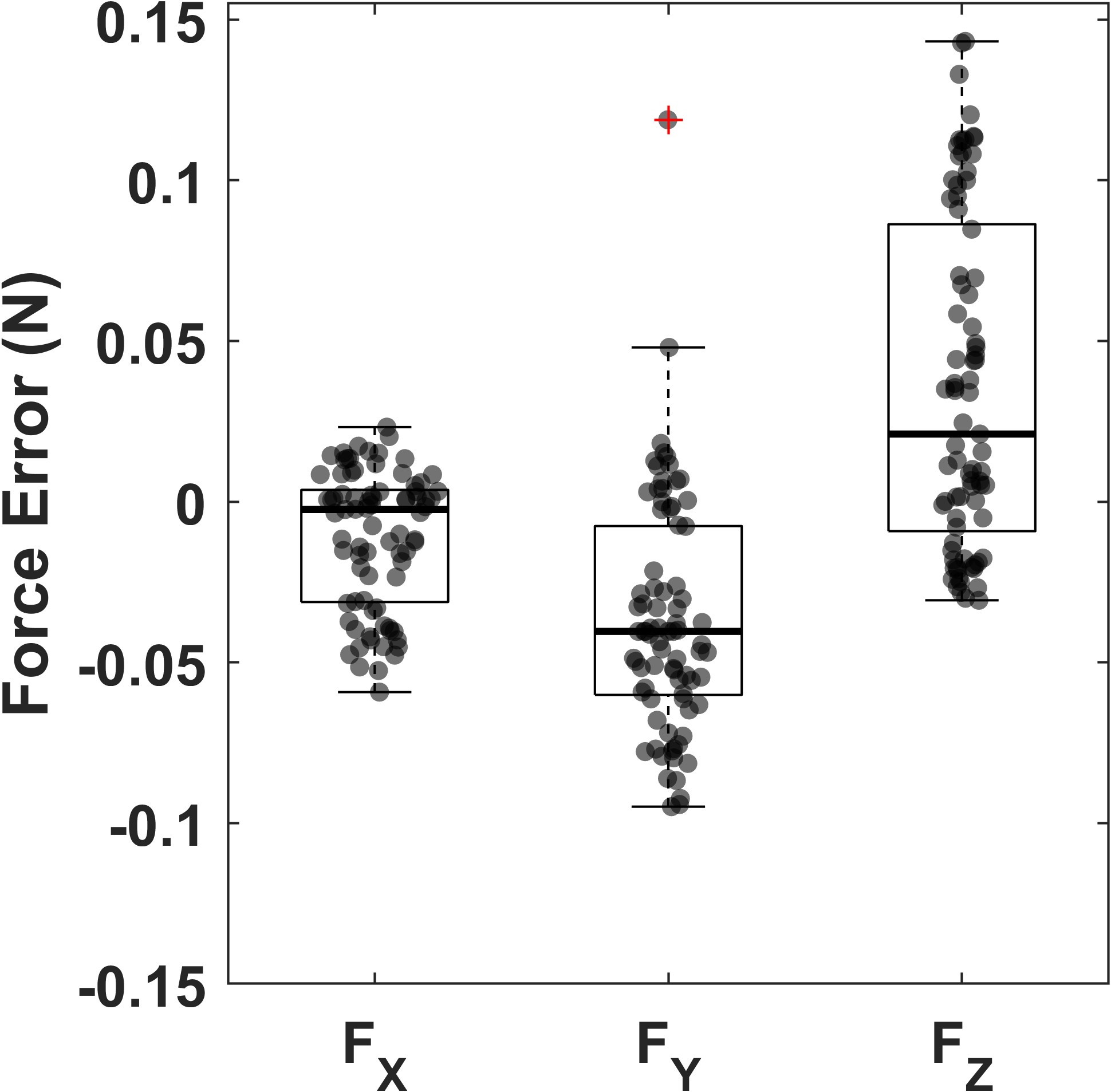

Supplement: Supplementary file 3 [file DataSheet2.zip › Appendix figures/Figure A C.jpeg]

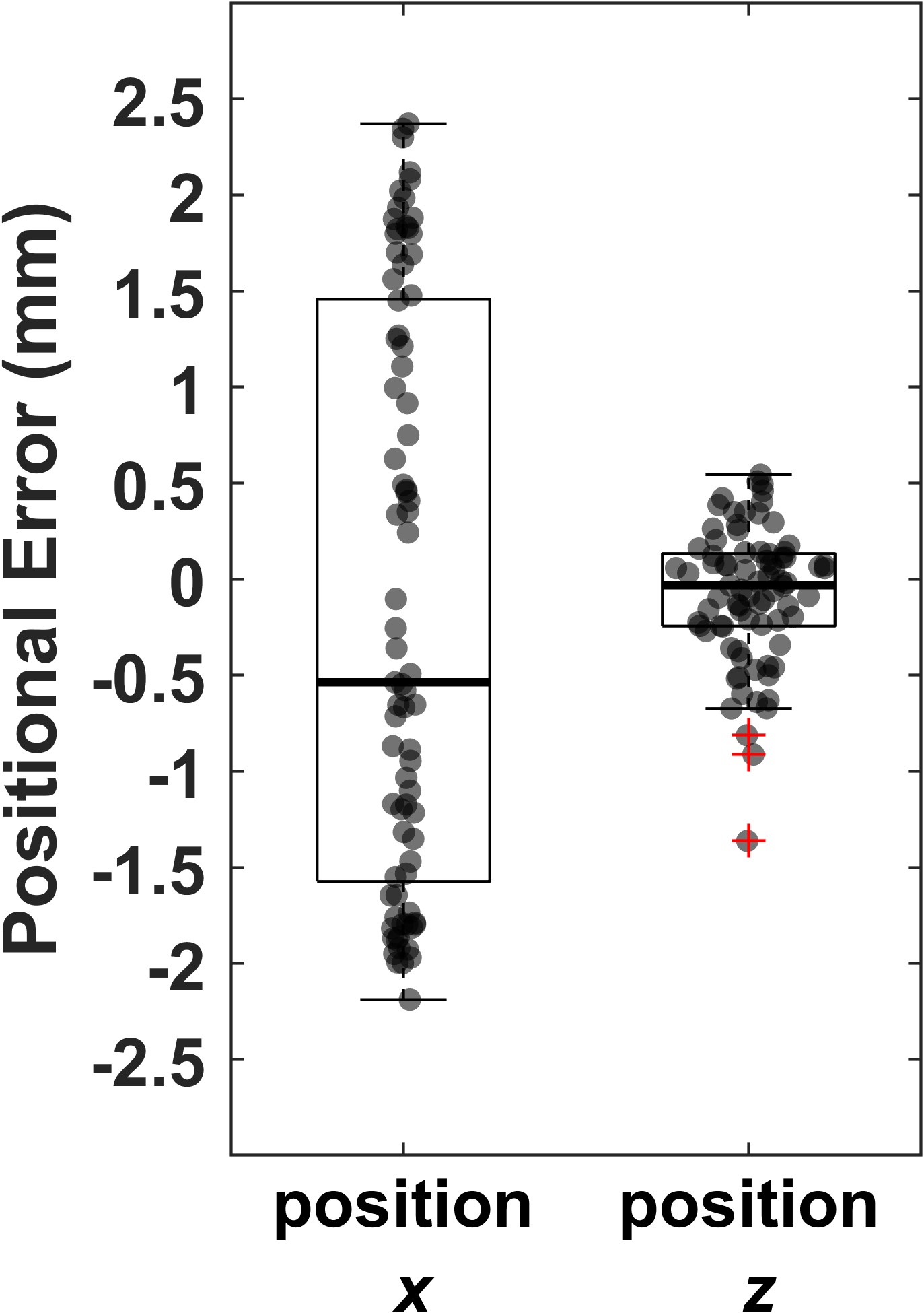

Supplement: Supplementary file 3 [file DataSheet2.zip › Appendix figures/Figure A D.jpeg]

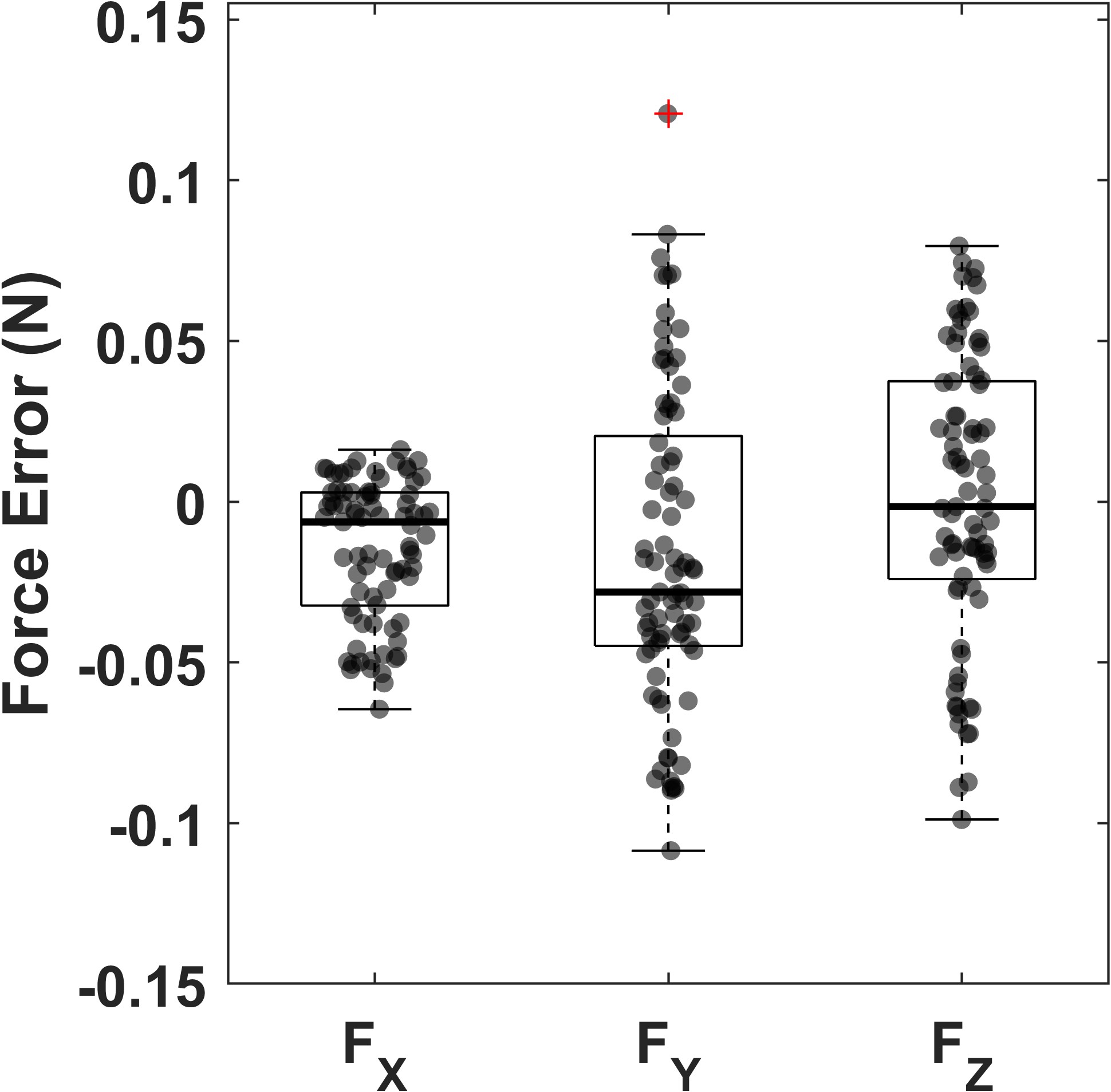

Supplement: Supplementary file 3 [file DataSheet2.zip › Appendix figures/Figure A E.jpeg]

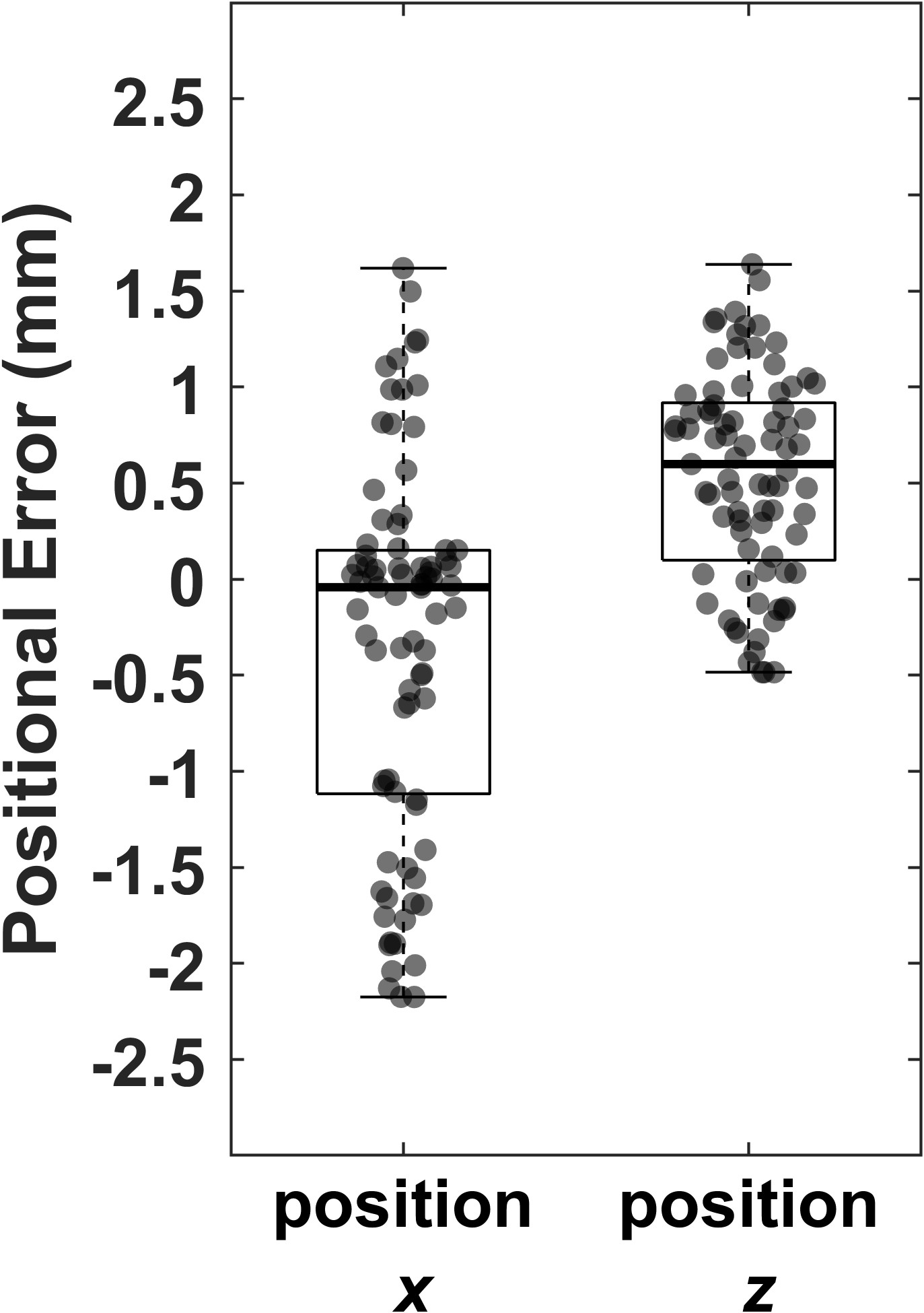

Supplement: Supplementary file 3 [file DataSheet2.zip › Appendix figures/Figure A F.jpeg]

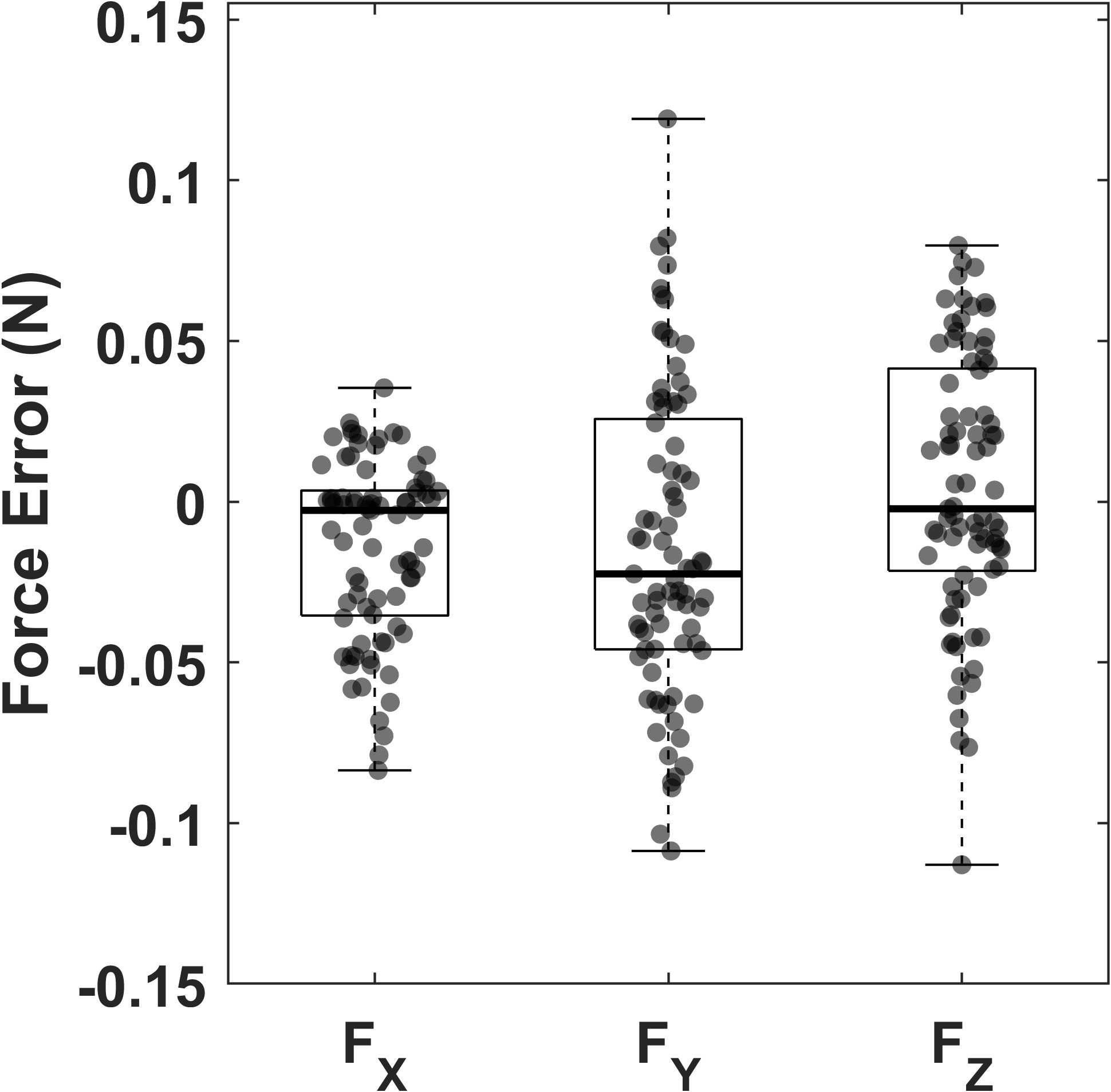

Supplement: Supplementary file 3 [file DataSheet2.zip › Appendix figures/Figure A G.jpeg]

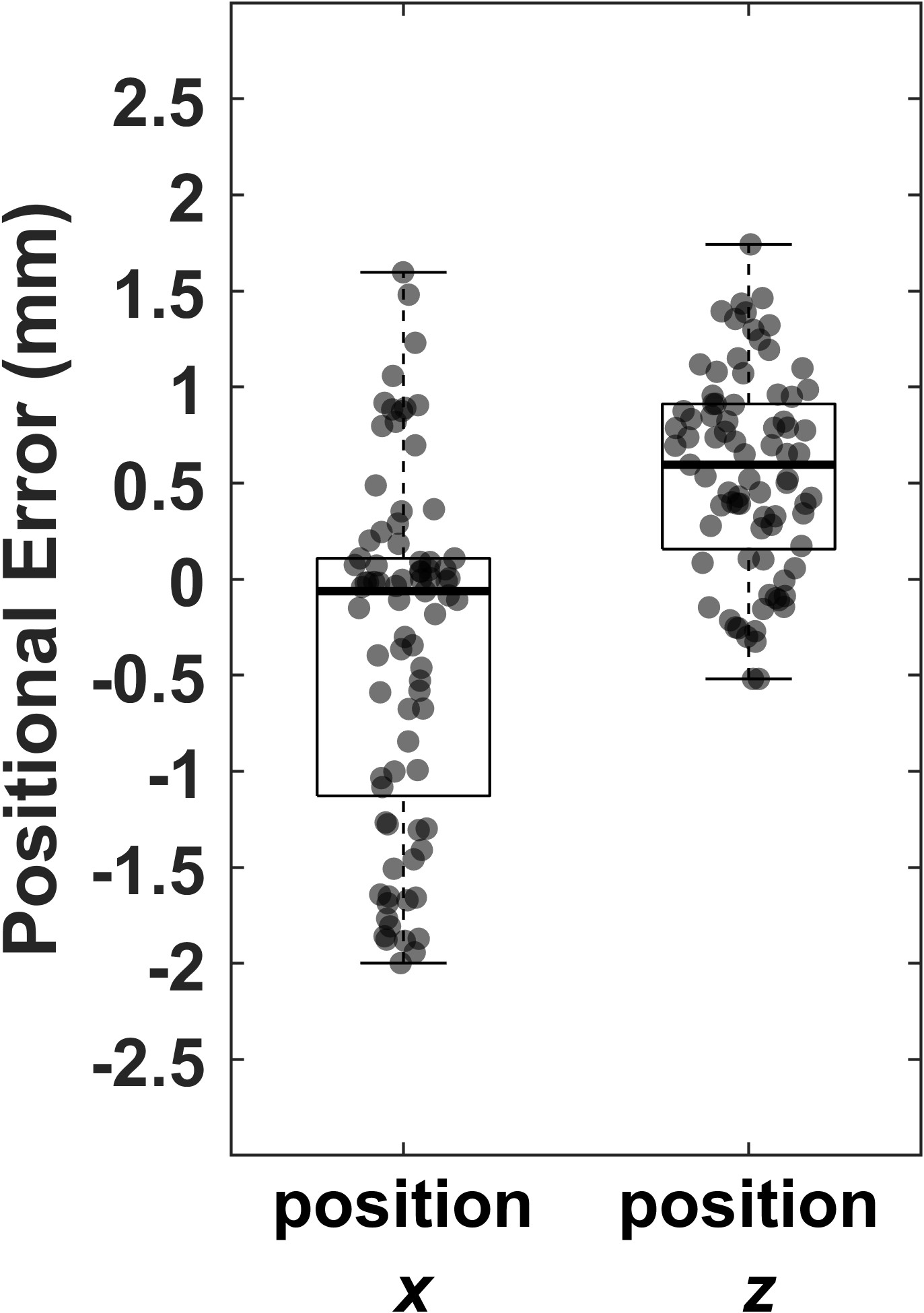

Supplement: Supplementary file 3 [file DataSheet2.zip › Appendix figures/Figure A H.jpeg]

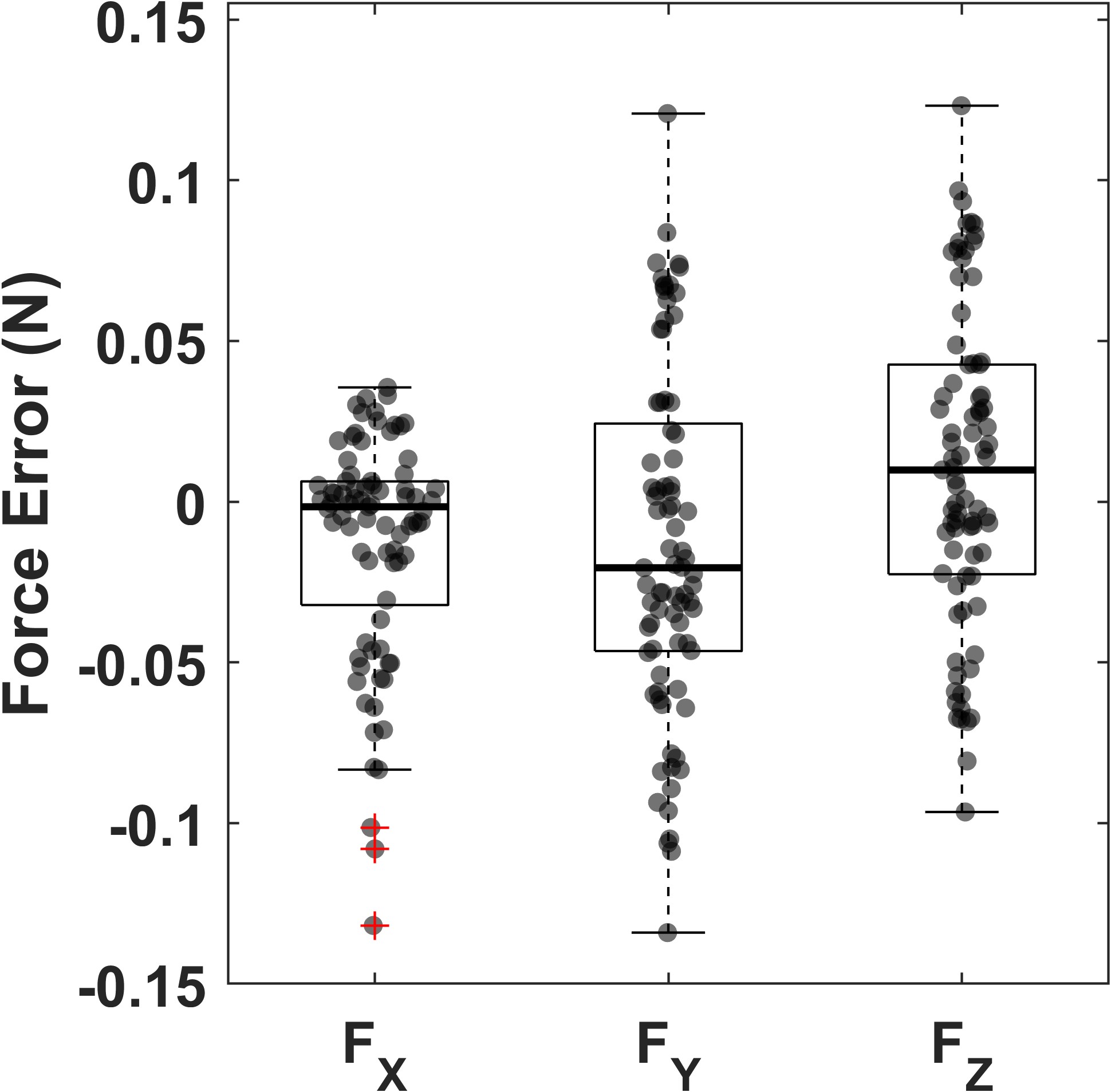

Supplement: Supplementary file 3 [file DataSheet2.zip › Appendix figures/Figure A I.jpeg]

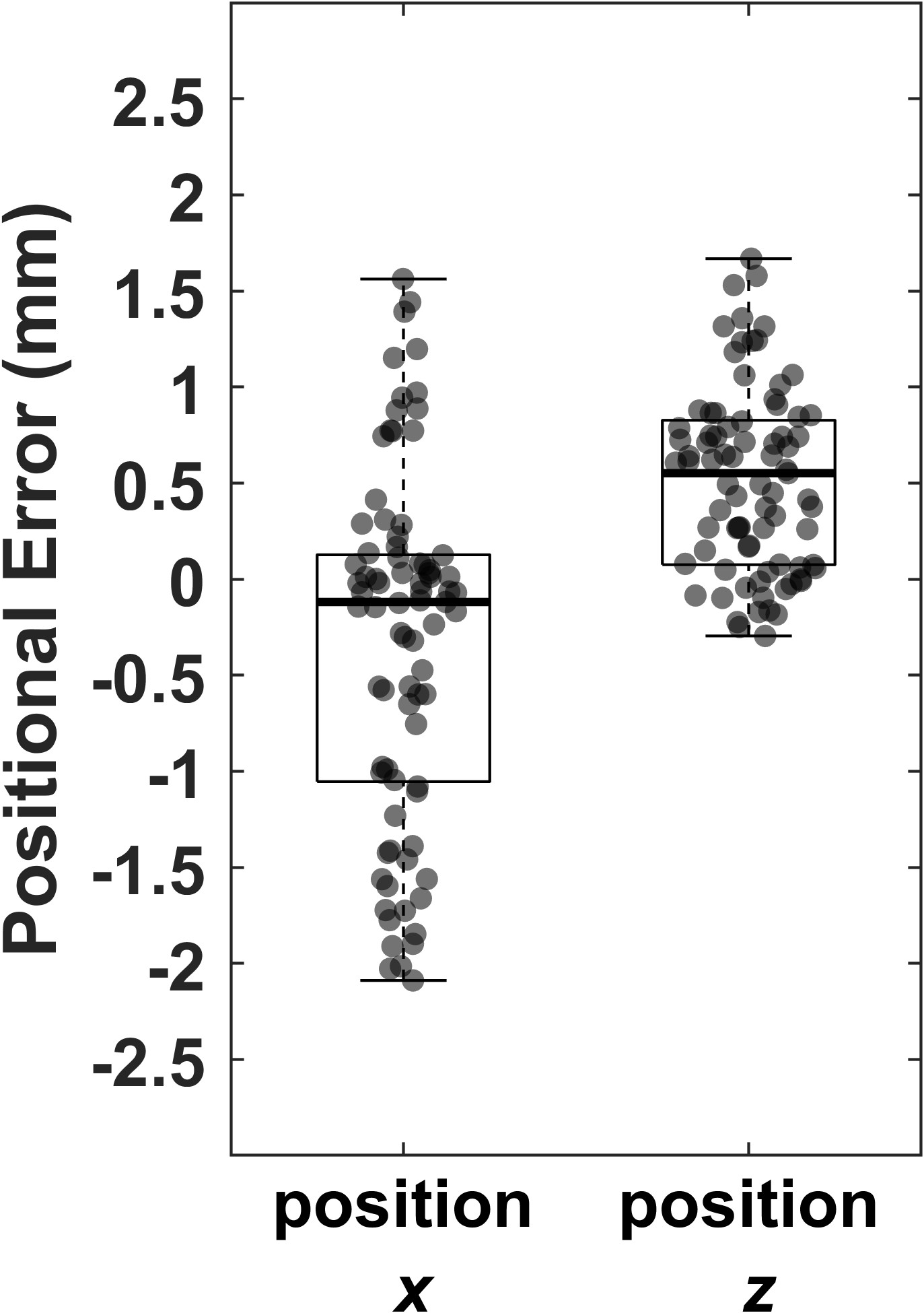

Supplement: Supplementary file 3 [file DataSheet2.zip › Appendix figures/Figure A J.jpeg]
